# A Rare Benign Mimic of Gastrointestinal Stromal Tumor: Gastric Plexiform Fibromyxoma Presenting With Iron-Deficiency Anemia and a Hypervascular Ulcerated Pyloric Mass

**DOI:** 10.7759/cureus.115543

**Published:** 2026-08-31

**Authors:** Dana B Abdo, Ahmed Ahmed, Hassan Hatab, Anas Hatab

**Affiliations:** 1 Internal Medicine, Royal Blackburn Teaching Hospital, Blackburn, GBR; 2 Emergency Medicine, University Hospitals Dorset NHS Foundation Trust, Bournemouth, GBR; 3 Gastroenterology, East Lancashire Hospitals NHS Trust, Blackburn, GBR; 4 Gastroenterology, Royal Blackburn Teaching Hospital, Blackburn, GBR

**Keywords:** acute care medicine, clinical pathology, general internal medicine, git endoscopy, imt

## Abstract

Gastric lesions with suspicious radiological and endoscopic features can remain diagnostically challenging when initial tissue sampling is benign or non-diagnostic. This case highlights the importance of correlating endoscopic appearance, cross-sectional imaging, endoscopic ultrasound (EUS) findings, histology, and multidisciplinary assessment when investigating a deeper gastric lesion.

A 28-year-old man was investigated for iron-deficiency anemia associated with intermittent abdominal pain and altered bowel habit. Laboratory investigations demonstrated microcytic iron-deficiency anemia, with a hemoglobin nadir of 95 g/L, below the reference range of 130-180 g/L, and markedly reduced iron stores. Colonoscopy, including terminal ileal and colonic biopsies, was normal. Gastroscopy identified an ulcerated polypoidal lesion in the antral/prepyloric region. Computed tomography (CT) demonstrated a 3.5 cm highly vascularized lesion within the pylorus, with no lymphadenopathy or distant lesions. Initial mucosal biopsies showed benign foveolar hyperplasia but contained no submucosal tissue, limiting assessment of a deeper lesion. EUS demonstrated an approximately 4 cm lesion arising from the muscularis propria with ulceration; however, fine-needle biopsy was hemorrhagic and non-diagnostic. Repeat tissue sampling showed benign hyperplastic-type gastric mucosa without evidence of gastrointestinal stromal tumor (GIST), neuroendocrine tumor, dysplasia, or malignancy in the available tissue. Owing to the lesion’s transmural nature and discordant clinicoradiological findings, it was considered unsuitable for endoscopic submucosal dissection (ESD). The patient underwent robotic-assisted distal gastrectomy with Roux-en-Y reconstruction. Specialist Liverpool pathology review of the distal/subtotal gastrectomy specimen reported a final diagnosis compatible with gastric plexiform fibromyxoma.

This case highlights that benign superficial biopsies and non-diagnostic EUS-guided sampling should not be considered definitive when CT and EUS suggest a deeper transmural gastric lesion. Adequate tissue assessment and specialist pathology review are essential when endoscopic, radiological, and histological findings are discordant.

## Introduction

Gastric subepithelial lesions may be difficult to diagnose because benign and malignant tumors can share overlapping endoscopic and radiological appearances. A vascular or ulcerated gastric lesion arising from the muscularis propria is particularly concerning for a gastric mesenchymal tumor such as gastrointestinal stromal tumor (GIST) [1]. Neuroendocrine tumor and malignancy may also be considered depending on the lesion’s appearance, location, and clinical context.

Plexiform fibromyxoma is a rare benign mesenchymal tumor of the stomach, previously described as plexiform angiomyxoid myofibroblastic tumor [2]. It most often involves the gastric antrum or pyloric region [3]. Histological recognition depends on identifying the characteristic plexiform growth pattern within a myxoid or angiomyxoid stroma, together with appropriate immunohistochemical correlation to distinguish it from GIST [2-5]. Its rarity and overlap with other gastric subepithelial tumors mean that diagnosis can be challenging, particularly when preoperative biopsies are superficial or non-diagnostic [4,5].

We report a case of gastric plexiform fibromyxoma presenting with iron-deficiency anemia and an ulcerated hypervascular pyloric lesion that mimicked GIST, neuroendocrine tumor, or malignancy before the final reported diagnosis was made on specialist review of the gastrectomy specimen.

## Case presentation

A 28-year-old man was referred to gastroenterology for investigation of iron-deficiency anemia. He reported intermittent abdominal pain over several weeks, mainly affecting the upper abdomen and sometimes radiating more widely. He also described altered bowel habit, with loose stools predominating at times and intermittent constipation. His weight was stable, and there was no reported family history of inflammatory bowel disease or gastrointestinal malignancy. He was taking oral iron replacement.

Initial laboratory investigations showed a microcytic iron-deficiency pattern. His hemoglobin reached a nadir of 95 g/L, below the reference range of 130-180 g/L. Mean corpuscular volume was reduced at 71.5 fL, below the reference range of 76.0-100.0 fL, and mean corpuscular hemoglobin was reduced at 17.9 pg, below the reference range of 27.0-32.0 pg. Iron studies demonstrated markedly reduced ferritin at 6 µg/L, below the reference range of 22-322 µg/L, serum iron of 2.7 µmol/L, below the reference range of 11.6-31.3 µmol/L, and transferrin saturation of 3.3%, below the reference range of 20.0%-50.0%. Vitamin B12 and folate were not deficient. Coeliac serology was negative, and inflammatory markers were not elevated.

Bidirectional endoscopy was arranged. Colonoscopy reached the terminal ileum and did not identify a lower gastrointestinal source of bleeding or inflammation. Terminal ileal and random colonic biopsies were normal.

Gastroscopy demonstrated an ulcerated polypoidal lesion in the antral/prepyloric region. Initial biopsies from the antral lesion showed polypoidal antral mucosa with foveolar hyperplasia, edema, mild chronic inflammation, and congestion. There was no dysplasia or carcinoma. However, no submucosal tissue was present in the biopsy. The pathology report advised clinicoradiological correlation, noting that the biopsy might not be representative if the lesion was submucosal.

Contrast-enhanced computed tomography (CT) of the thorax, abdomen, and pelvis demonstrated a 3.5 cm highly vascularized lesion within the pylorus. There was no lymphadenopathy and no focal liver or lung lesion. The radiological differential diagnosis included neuroendocrine tumor, GIST, or malignant tumor, although further correlation with the endoscopic appearance and histology was recommended. The lesion is shown on axial contrast-enhanced CT images (Figure 1).

**Figure 1:**
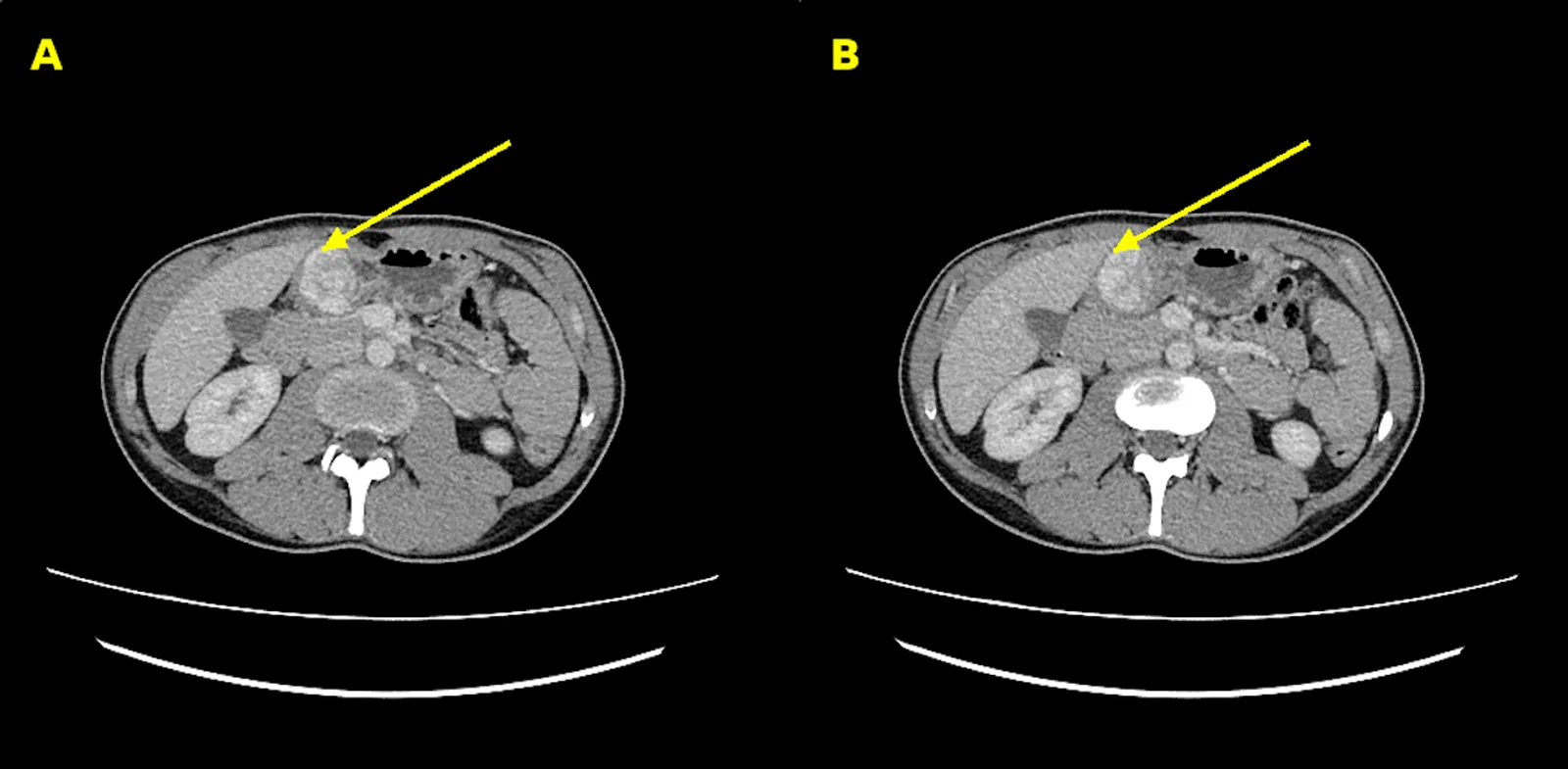
Axial contrast-enhanced CT images demonstrating a hypervascular pyloric/antral lesion Axial contrast-enhanced CT images demonstrating a well-defined hypervascular lesion in the pyloric/antral region, indicated by arrows. (A) Representative slice showing the lesion most clearly. (B) Adjacent axial slice further demonstrating the lesion in relation to the pyloric/antral region.

The case was discussed at the upper gastrointestinal multidisciplinary meeting, and further tissue assessment was arranged. Endoscopic ultrasound (EUS) demonstrated an approximately 4 cm lesion arising from the muscularis propria, with ulceration in the antrum. Fine-needle biopsy was performed, but the sample was hemorrhagic and suboptimal, showing only scant inflammatory cells and degenerated cells. It was reported as non-diagnostic.

Repeat endoscopic assessment demonstrated a large polypoid prepyloric/antral lesion with central ulceration. Further biopsy showed benign polypoidal gastric mucosal fragments with hyperplastic features. Focal intestinal metaplasia was present in occasional crypts. The lamina propria was edematous with congested vessels and a mixed inflammatory infiltrate. Immunohistochemistry performed on the available material did not show evidence of GIST or neuroendocrine tumor. There was no dysplasia, *Helicobacter pylori*, or malignancy in the sampled tissue.

Despite the reassuring mucosal biopsies, the overall findings remained discordant. The lesion was ulcerated, highly vascularized on CT, and appeared to arise from the muscularis propria on EUS. The multidisciplinary team summary also described ulcer-associated bleeding from the lesion that did not require endoscopic intervention. The lesion was therefore not considered to be adequately explained by a superficial hyperplastic gastric polyp.

Further upper gastrointestinal multidisciplinary review concluded that the lesion was approximately 3.5 cm, transmural, and not suitable for endoscopic submucosal dissection (ESD). The patient subsequently underwent robotic-assisted distal gastrectomy with Roux-en-Y reconstruction and intraoperative oesophagogastroduodenoscopy (OGD). Postoperative pathology reported negative GIST and epithelial markers, and the case was referred for specialist pathology review. Liverpool second-opinion pathology review of the distal/subtotal gastrectomy specimen reported a final diagnosis compatible with gastric plexiform fibromyxoma. Molecular testing was pending at the time of multidisciplinary team discussion.

## Discussion

Gastric plexiform fibromyxoma is an uncommon benign mesenchymal tumor that most frequently arises in the antrum or pyloric region of the stomach [2,3]. It has also been described as a plexiform angiomyxoid myofibroblastic tumor, reflecting its characteristic plexiform growth pattern and myxoid or angiomyxoid stroma [2]. Although generally regarded as benign, its clinical, endoscopic, radiological, and pathological features may overlap with other gastric subepithelial tumors, particularly GIST [3-5].

This case demonstrates the diagnostic difficulty of gastric plexiform fibromyxoma when it presents as an ulcerated hypervascular pyloric lesion. The patient initially presented with iron-deficiency anemia, supported by microcytosis and reduced iron stores. Colonoscopy and ileocolonic biopsies did not identify a lower gastrointestinal source. In this context, the ulcerated pyloric/antral lesion, with bleeding described on endoscopic review, provided a plausible explanation for chronic occult upper gastrointestinal blood loss.

The lesion closely mimicked GIST. It was located in the antral/prepyloric region, measured approximately 3.5-4 cm, was highly vascularized on CT, and appeared to arise from the muscularis propria on EUS. These features appropriately raised concern for a gastric mesenchymal tumor, particularly GIST. Neuroendocrine tumor and malignancy were also considered because of the lesion’s vascularity, ulceration, and pyloric location. Previous reports have similarly highlighted that plexiform fibromyxoma may mimic GIST clinically, endoscopically, radiologically, and intraoperatively [3-5].

The central diagnostic pitfall in this case was the mismatch between suspicious imaging and reassuring tissue samples. Initial biopsies showed benign foveolar and hyperplastic-type mucosal changes, but no submucosal tissue was included. This is an important limitation because superficial mucosal biopsies may sample only reactive surface change overlying a deeper subepithelial lesion. Similar diagnostic pitfalls have been described in gastric plexiform fibromyxoma, where limited biopsy material may not capture the diagnostic architecture of the lesion [4,5]. This represents an important form of sampling error in subepithelial or transmural gastric lesions, where the superficial mucosa may not reflect the underlying pathology [4,5].

EUS-guided sampling also failed to provide a diagnosis. Although EUS helped localize the lesion to the muscularis propria, fine-needle biopsy was hemorrhagic and non-diagnostic. Repeat biopsy showed benign mucosal fragments without evidence of GIST, neuroendocrine tumor, dysplasia, or malignancy in the available material. Therefore, the absence of malignancy in the biopsies did not resolve the diagnostic concern because the lesion’s behavior and morphology suggested a deeper transmural process. This supports the need for clinicoradiological correlation when biopsy results do not explain the endoscopic or imaging appearances [4,5].

Management was driven by the overall clinicoradiological picture rather than by superficial biopsy results alone. The lesion was considered transmural and unsuitable for ESD. Surgical resection provided adequate tissue for definitive pathological assessment. Specialist review of the distal/subtotal gastrectomy specimen from Liverpool reported a final diagnosis compatible with gastric plexiform fibromyxoma.

The distinction from GIST is clinically important because these entities differ in expected behavior and management [1,3-5]. GISTs commonly express KIT proto-oncogene receptor tyrosine kinase (KIT), also assessed immunohistochemically as cluster of differentiation 117 (CD117), or discovered on GIST 1 (DOG1), whereas plexiform fibromyxoma typically lacks this immunophenotypic profile and requires recognition of its characteristic morphology with appropriate pathological correlation [1,3-5]. In this case, the available preoperative tissue did not show evidence of GIST or neuroendocrine tumor, but the diagnosis could not be confidently resolved until the resection specimen underwent specialist review.

The key lesson from this case is that benign superficial biopsies should not end the diagnostic process when imaging and EUS suggest a deeper ulcerated transmural lesion. Discordance between endoscopic appearance, radiology, EUS, and histology should prompt multidisciplinary discussion, recognition of sampling limitations, and consideration of adequate tissue diagnosis. Gastric plexiform fibromyxoma should be considered in the differential diagnosis of antral or pyloric hypervascular subepithelial lesions that mimic GIST, particularly when initial biopsies are benign or non-diagnostic [3-5].

## Conclusions

Gastric plexiform fibromyxoma is a rare benign gastric mesenchymal tumor that can closely mimic GIST, particularly when it presents as an ulcerated hypervascular pyloric lesion arising from a deeper gastric layer. In this case, iron-deficiency anemia led to the discovery of a suspicious pyloric/antral mass, while repeated mucosal biopsies and EUS-guided sampling were either non-representative or non-diagnostic. This highlights the importance of not relying solely on benign superficial biopsies when CT and EUS findings suggest a deeper transmural lesion. Multidisciplinary review, surgical tissue diagnosis, and specialist gastrointestinal pathology input were central to establishing the final diagnosis of gastric plexiform fibromyxoma.
